# Reassessment of Miocene putative coprolites from Poland suggests a biogenic origin and probable squamate (reptilian) producers

**DOI:** 10.7717/peerj.21755

**Published:** 2026-09-29

**Authors:** Tomasz Brachaniec, Dorota Środek, Michał Bugajski, Grzegorz Sadlok, Bartosz Czader, Adam Danielak, Oleksandr Kovalchuk

**Affiliations:** 1 University of Silesia in Katowice, Faculty of Natural Sciences, Sosnowiec, Poland; 2 Fossil Amateur Club “Inkluzja”, Łódź, Poland; 3 Municipal Zoological Garden in Łódź, Łódź, Poland; 4 National Museum of Natural History of the National Academy of Sciences of Ukraine, Kyiv, Ukraine; 5 Department of Palaeozoology, Faculty of Biological Sciences, University of Wrocław, Wrocław, Poland

**Keywords:** Coprolites, Faeces, Lizards, Snakes, Miocene, Kleszczów Graben, Poland

## Abstract

Additional ferruginous excrement-shaped masses interpreted as putative coprolites were recovered from Miocene deposits of the Kleszczów Graben, central Poland. Earlier studies favored an abiotic origin, but our reassessment provides evidence supporting a biogenic interpretation. Key indicators include their consistent size and morphology, fine surface striations, and the presence of hair-like inclusions. These features distinguish the relatively homogeneous, elongated, and variably compressed forms from morphotypes previously described in the Turów and Kleszczów areas (southwestern and central Poland, respectively). Comparisons with modern faeces suggest that squamates (lizards and/or snakes) were the most probable producers, consistent with reptilian remains documented from the Miocene deposits of Bełchatów. Alternative vertebrate producers and non-biogenic explanations appear less consistent with the observed characteristics. Our findings highlight the palaeobiological processes involved in the formation of these structures and provide new insights into the faunal composition and ecological dynamics of Miocene ecosystems in central Poland.

## Introduction

Ferruginous, excrement-shaped masses from Neogene deposits in Poland have long been the subject of debate regarding their origin and possible producers. Brachaniec et al. (2022) provided the first detailed description of such structures from the Turów region in southwestern Poland, cautiously designating them as “alleged” coprolites due to uncertainties in their formation. Based on morphology, the authors tentatively attributed their production to testudinoid turtles or snakes, while noting that lizards, crocodiles, or abiotic processes could not be entirely excluded. A subsequent synthesis by Brachaniec et al. (2025) expanded the geographic and stratigraphic scope to include marine Oligocene–Miocene sediments of southeastern and southern Poland, and terrestrial Miocene deposits from southwestern and central regions. Within the Miocene successions of Turów and the Kleszczów Graben, five morphotypes were recognized: oval, sinusoidal, elongated, S-shaped, and irregular. Most specimens consisted of sideritic masses with rough, coalified surfaces, fine external striations, and internal filamentous inclusions. These features supported their interpretation as coprolites, with snakes considered the most likely producers. The filamentous inclusions further suggested dietary associations with small mammals, such as squirrels (Sciuridae) or bats (Chiroptera).

Building on these observations, the present study documents a previously undescribed morphological variant from the Miocene deposits of the Bełchatów lignite mine, situated within the Kleszczów Graben in central Poland. Integrated morphological and microstructural analyses support their interpretation as coprolites of probable biogenic origin. We propose that squamates (lizards and/or snakes) were most likely producers, thereby extending the taxonomic and ecological framework established in earlier studies. This contribution refines our understanding of faunal composition, trophic interactions, and depositional environments in Miocene ecosystems of central Poland.

## Geological setting and stratigraphy

The Kleszczów Graben is a major tectonic structure in central Poland, extending over 80 km in length and up to 3 km in width (Figs. 1A, 1B, 1C). Its basement comprises Permian, Jurassic, and Cretaceous sediments, overlain by younger strata (Olchowy, Krajewski & Felisiak, 2019). The graben originated in the Paleocene and underwent three phases of tectonic deformation (Krzyszkowski, 1989). Regional uplift during the Late Oligocene (Chattian) accelerated sedimentary processes within the basin. The oldest Miocene deposits consist of sands, silts, clays, and lignite, with the earliest lignite complex overlying these siliciclastic units (Fig. 1D; Czarnecki, Frankowski & Kuszneruk, 1992).

**Figure 1:**
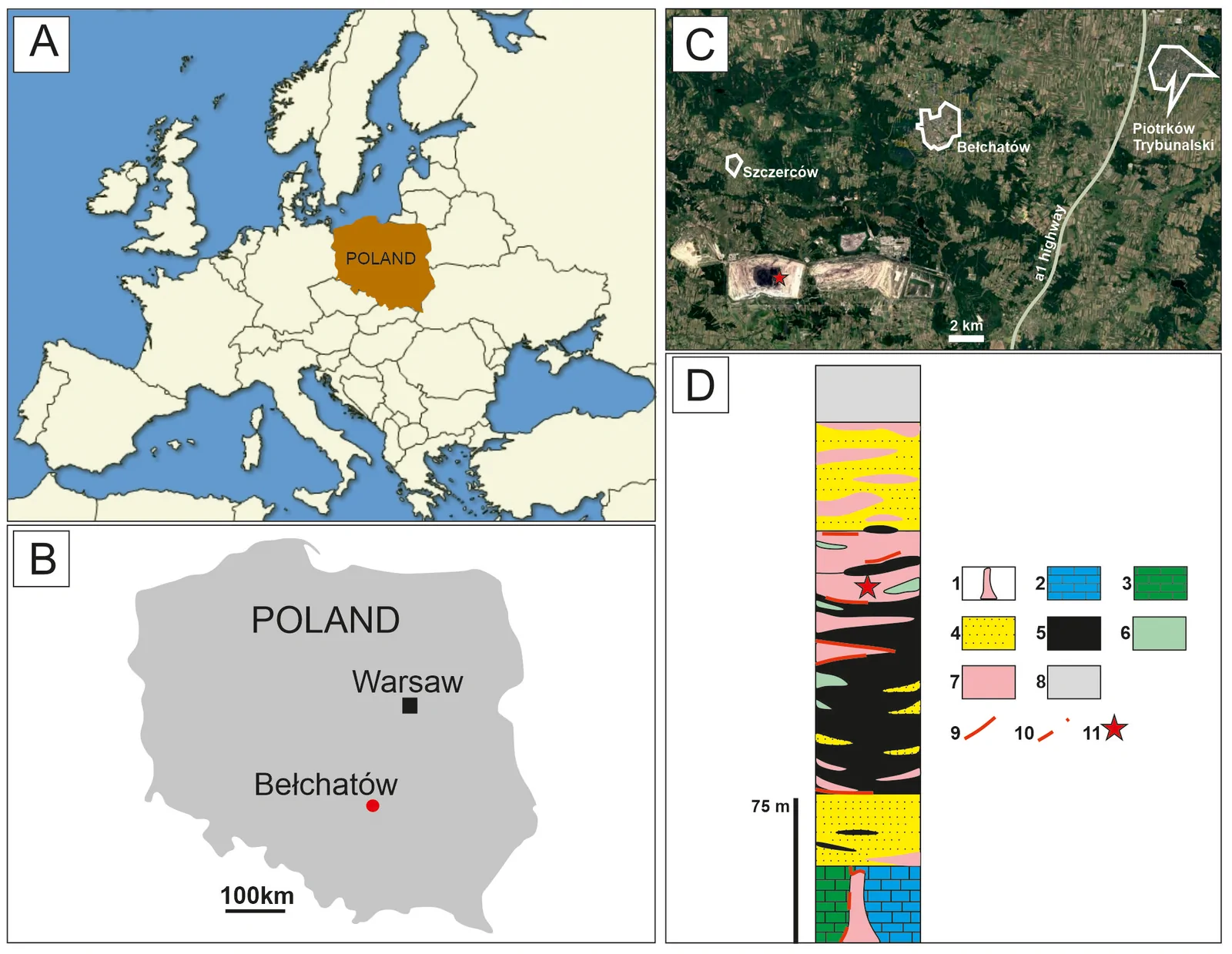
Investigated area. (A) Position of Poland within Europe. (B) Location of the Bełchatów outcrop in Poland. (C) Locality of the investigated outcrop in the Kleszczów Graben , marked with a red star. (D) Lithological profile of the Bełchatów section (modified after Widera, Klesk & Urbanski, 2024): 1–Permian/Cenozoic clays, gypsum cap; 2–Jurassic carbonates; 3–Cretaceous marlstones; 4–Neogene sands; 5–Neogene lignite; 6–gyttja and lacustrine chalk; 7–clays, silts, mud; 8–Quaternary deposits; 9–fault; 10–tuff; 11–coprolites location.

The Lower Miocene lignite coal complex is interbedded with sands, clays, lacustrine chalk, flint, sandstones, and paratonsteins, reflecting a heterogeneous depositional environment (Widera, Klesk & Urbanski, 2024). Middle Miocene strata comprise clay–carbonate and clay–sand complexes up to 150 m thick, rich in plant remains, vertebrate fossils, and coprolites (Brachaniec et al., 2022; Brachaniec et al., 2025; Brachaniec, Bugajski & Kovalchuk, 2026; Górka et al., 2025; Salamon, Bugajski & Degórski, 2025; Salamon et al., 2026). These assemblages indicate swampy and marshland habitats dominated by rushes, alongside forests and braided river systems. Coprolites provide direct evidence of local faunal activity, while surface features such as desiccation cracks suggest periodic subaerial exposure during deposition.

## Materials and Methods

The specimens interpreted as putative coprolites were recovered from Miocene deposits of the Kleszczów Graben, specifically from the Bełchatów Brown Coal Mine in central Poland. All coprolite-like forms, associated vertebrate remains (teeth, osteoderms), and previously recognized morphotypes derive from a single stratigraphic horizon approximately 40 cm thick. Sediment variability within this interval (gyttja, lacustrine chalk, sands, muds, clays, thin lignite layers, clay–coal, and clayey sands) reflects lateral facies changes rather than multiple stratigraphic levels. Coprolites occur consistently within this horizon, occasionally in direct association with vertebrate remains.

Approximately 150 kg of raw sediment was collected over several months. Samples were washed in water and processed using a two-sieve system: a two mm mesh retained larger fractions, while a 0.35 mm mesh retained smaller particles. Larger particles were examined under a magnifying glass and, when necessary, remacerated. The smaller fraction was dried and examined under a PZO MST 131 microscope at 10 × magnification. All specimens are curated at the Institute of Earth Sciences, Faculty of Natural Sciences, University of Silesia in Katowice, Poland, where they are stored under the registration summary number GIUS 10–3796/Sz.

Fieldwork was conducted with the permission of the site owners/operators, which was communicated verbally to one of the co-authors (M. Bugajski). Access to the study area was granted on site, and no formal permit number was issued.

### Optical microscopy and thin-sectioning

Thin sections were prepared at the grindery of the Institute of Earth Sciences. Fragile coprolite samples were embedded in Araldite epoxy resin, sectioned, and mounted on glass slides. Polishing was performed with silicon carbide and aluminium oxide abrasives. Sections (∼30 µm thick) were cut from multiple parts of each specimen to document internal variability. Preliminary observations were made with a Leica SZ-630T stereomicroscope and Nikon Eclipse E100 light microscope. High-resolution imaging was conducted using an Olympus BX53M polarizing microscope equipped with an Olympus UC90 camera and halogen light source. This setup enabled detailed documentation of internal structures. All analyses were performed at the Institute of Earth Sciences, ensuring high standards of scientific accuracy.

### Scanning electron microscopy

The coprolite matrix and inclusions were analyzed using a Phenom XL desktop scanning electron microscope (Phenom World, Thermo Fisher Scientific, Netherlands), equipped with an energy-dispersive X-ray spectroscopy (EDS) detector and secondary electron detector (SED). Observations were conducted under low vacuum conditions at 15 kV. Samples were not coated.

### Extant excrements

Modern excrement samples were collected from animals at the Municipal Zoological Garden in Łódź, Poland, and from individuals in natural habitats. Observations at the zoo were conducted by staff member AD with institutional permission. Additional comparative data were incorporated from Brachaniec et al. (2022), Brachaniec et al. (2025) and direct field observations. Samples included excrements from fish, reptiles, birds, and mammals, with emphasis on taxa represented in Miocene strata of Poland and neighboring regions. All samples were photographed and documented for comparative analysis.

## Results

### Coprolite morphology

A total of 23 specimens, interpreted as putative coprolites, were recovered. All belong to the three most dominant types (elongated, less or more squeezed), characterized by elongation and variable compression. The specimens display relatively uniform coloration, ranging from pale orange and light brown to greenish-red and burgundy (Figs. 2A–2F). Lengths range from 35 to 142 mm, with widths between 12 and 44 mm. A detailed summary of measurements is provided in Table S1.

**Figure 2:**
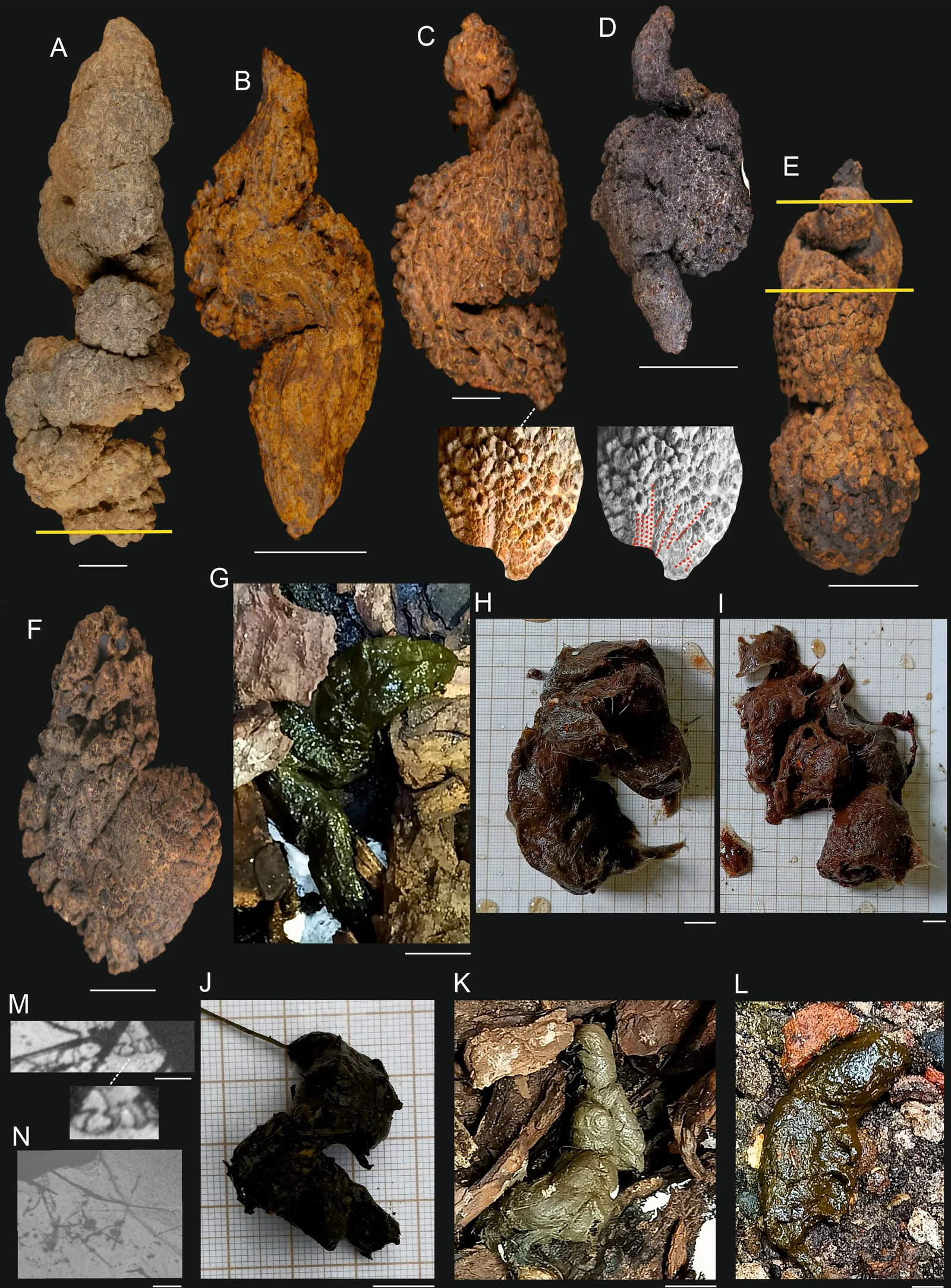
Studied material interpreted as probable coprolites and compared to them modern faeces. (A–F) Miocene forms from the Kleszczów Graben, central Poland. (A) GIUS 10–3796/Sz /c/1. (B) GIUS 10–3796/Sz /c/2. (C) GIUS 10–3796/Sz /c/3. (D) GIUS 10–3796/Sz /c/4. (E) GIUS 10–3796/Sz /c/5. (F) GIUS 10–3796/Sz /c/6. Coprolite C with striate texture. (G–L) Excrements of modern reptiles: (G, J) *Iguana delicatissima*; (H, I) *Acrantophis madagascariensis*; (K) *Chilabothrus subflavus*; (L) *Manouria emys*. (M, N) Hair-like structures observed in coprolite A (thin section marked with yellow line) under optical microscopy. Scale bars: one cm (A–L), 100 µm (M, N). Yellow lines in Fig. 2E indicate thin section cuts (see also Fig. 5).

### Accompanying fauna

Field investigations in Poland yielded a diverse assemblage of microfossils and macrofossils associated with the coprolites. Mollusk remains were present in all samples, with faunal composition varying by sediment type. Light lacustrine chalk contained relatively sparse microfauna, whereas grey clayey gyttja and darker, coal-rich sediments (with xylite fragments and detritus) were particularly rich in fossils. These facies included gastropods, bivalves, ostracods, and algae. Vertebrate remains were also recovered, notably small mammals belonging to Lagomorpha (Leporidae), Eulipotyphla (Talpidae), and Chiroptera. Additional vertebrate material, totaling ∼200 specimens, is summarized in Figs. 3–4 and Table S2 (see Brachaniec et al., 2025 for comparative data). The following Orders were identified: Esociformes, Anura, Crocodylia, Squamata, Eulipotyphla, Erinaceomorpha, Chiroptera, Lagomorpha, Rodentia. Among the collected material, teeth of crocodiles of the genus *Diplocynodon* sp. (Figs. 3B1–3B2; 4O) and a tooth with pleurodont dentition, likely belonging to a squamate (Figs. 3N1–3N3), were identified. Remains of frogs (Fig. 4G1) and a probable varanoid tooth (Fig. 3A) were also found. Unidentified reptile and lizard teeth are shown in Figs. 4F1–4F2, Figs. 4H1–4H2, Figs. 4I1–4I2, and Figs. 4J1–4J3.

**Figure 3:**
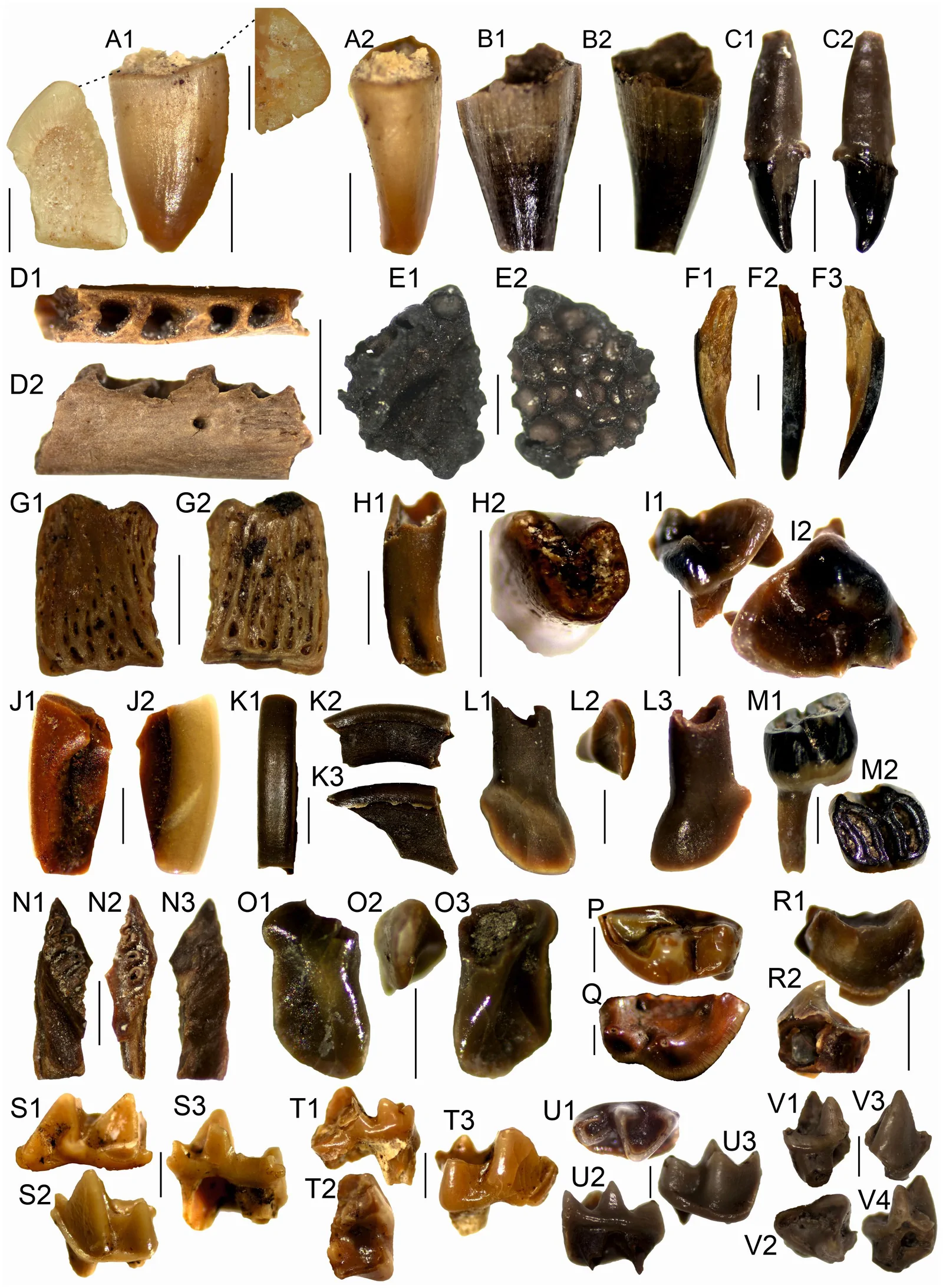
Selected vertebrate remains from Bełchatów. (A) Reptilia indet A1, lingual, A2, distal view; GIUS 10–3796/Sz /b/1 (B) Crocodylia indet tooth: b1, lingual, B2, mesial view; GIUS 10–3796/Sz /b/2 (C) *Myotis* sp., left Cs: C1, lingual, C2, labial view; GIUS 10–3796/Sz /b/3 (D) Erinaceidae indet., right mandible: D1, occlusal, D2, buccal view; GIUS 10–3796/Sz /b/4 (E) ?Anura indet., bone fragment: E1, ventral, E2, dorsal view; GIUS 10–3796/Sz /b/5 (F) Rodentia indet., lower right incisor: F1, mesial, F2, labial, F3, distal view; GIUS 10–3796/Sz /b/6 (G) osteoderm: G1, dorsal, G2, ventral view; GIUS 10–3796/Sz /b/7 (H) Lagomorpha indet., right ?m2: H1, lingual, H2, occlusal view; GIUS 10–3796/Sz /b/8 (I) *Desmanella* sp., tooth, right ?p4: I1, mesial, I2, occlusal view; GIUS 10–3796/Sz /b/9 (J) Rodentia indet., upper incisor: J1, lingual, J2, distal view; GIUS 10–3796/Sz /b/10 (K) Rodentia indet., right upper incisor fragment: K1, labial, K2, mesial, K3, distal view; GIUS 10–3796/Sz /b/11 (L) ?Erinaceidae indet., tooth, ?i3: L1, lingual, L2, incisal, L3, labial view; GIUS 10–3796/Sz /b/12 (M) *Keramidomys* sp., tooth, right m1: M1, lingual, M2, occlusal view; GIUS 10–3796/Sz /b/13 (N) ? Squamata indet ., N1, medial, N2, ventral, N3, lateral view; GIUS 10–3796/Sz /b/14 (O) *Desmanella* cf. *stehlini*, tooth, ?i2: O1, labial, O2, incisal, O3, lingual view; GIUS 10–3796/Sz /b/15 (P) ?Plesiodimylinae indet., tooth fragment, ventral view; GIUS 10–3796/Sz /b/16 (Q) ?Plesiodimylinae indet., tooth fragment, ventral view; GIUS 10–3796/Sz /b/17 (R) Talpinae indet., molar tooth fragment; r1, labial; r2, distal view; GIUS 10–3796/Sz /b/18 (S) *Desmanella* cf. *engesseri*, tooth, left m2: S1, occlusal, S2, labial, S3, lingual view; GIUS 10–3796/Sz /b/19 (T) *Plesiosorex germanicus,* tooth, right m3: T1, lingual, T2, occlusal, T3, labial view; GIUS 10–3796/Sz /b/20 (U) * Desmanella* cf. *stehlini,* tooth, right m3: U1, occlusal, U2, lingual, U3, labial view; GIUS 10–3796/Sz /b/21 (V) Talpinae indet., tooth, p4: V1, lingual, V2, occlusal, V3, labial, V4, distal/lingual view; GIUS 10–3796/Sz /b/22. Scale bars: one mm.

**Figure 4:**
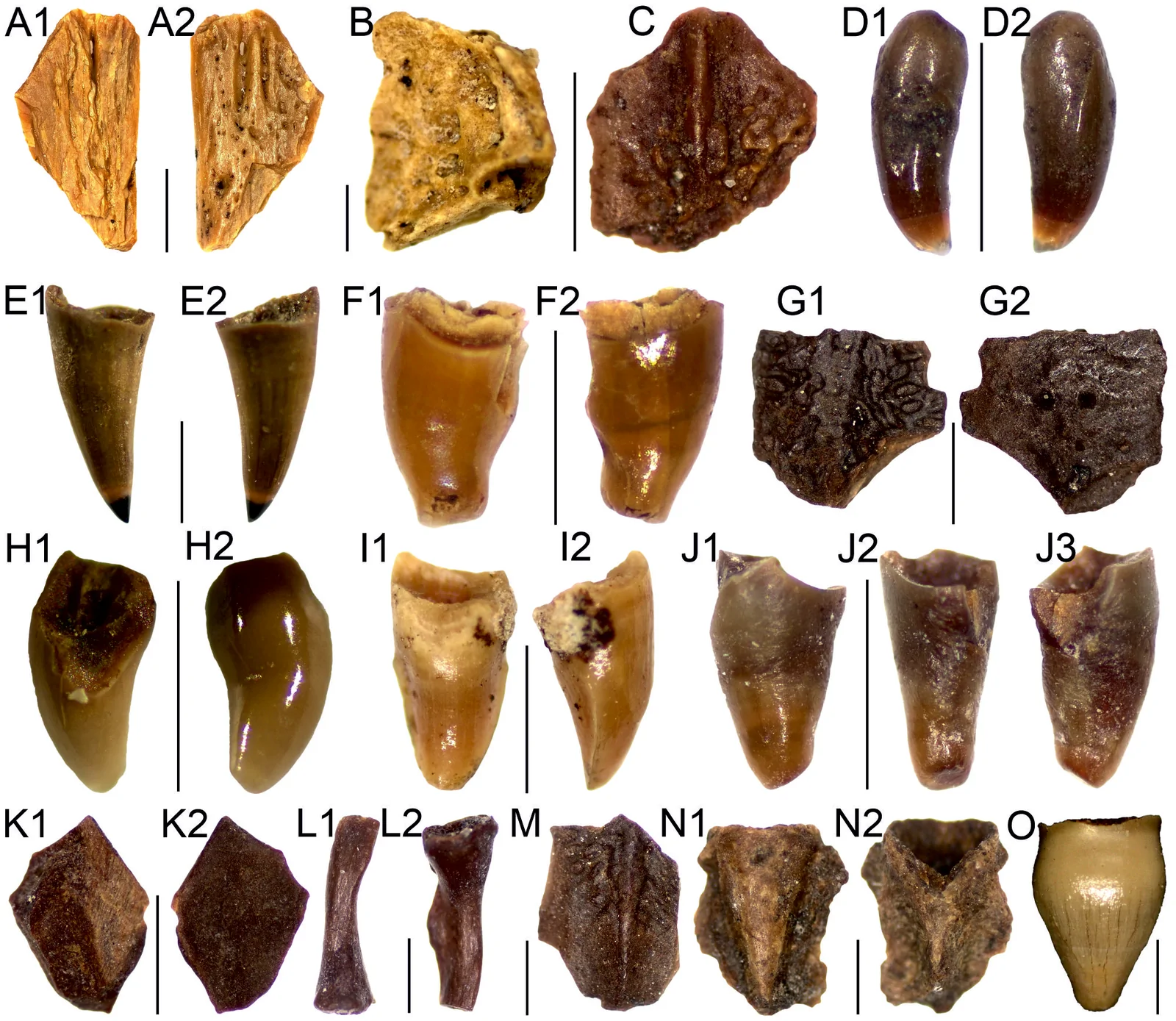
Additional vertebrate remains from Bełchatów. (A, B) ?Squamata, bone fragment: A1, A2, B1; GIUS 10–3796/Sz / b / 23-24. (C) Anguinae indet., osteoderm, external view; GIUS 10–3796/Sz / b / 25. (D) ?unidentified tooth: D1, lingual, D2, labial view; GIUS 10–3796/Sz / b / 26. (E) *Esox* sp., tooth: E1, lingual, E2 labial view; GIUS 10–3796/Sz / b / 27. (F) ?Reptilia, tooth: F1, lingual, F2, labial view; GIUS 10–3796/Sz / b / 28. (G) *Pelobates* sp., frontoparietal fragment: G1, dorsal, G2, ventral view; GIUS 10–3796/Sz / b / 29. (H) ?Reptilia, tooth: H1, lingual, H2, labial view; GIUS 10–3796/Sz / b / 30. (I) Reptilia indet., tooth: I1, lingual, I2, mesial view; GIUS 10–3796/Sz / b / 31. (J) ?Reptilia, tooth: J1, labial, J2, distal, J3, distalo-lingual view; GIUS 10–3796/Sz / b / 32. (K) bone fragment; GIUS 10–3796/Sz / b / 33. (L) Anura indet., left humerus: L1, dorsal, L2, anterior view; GIUS 10–3796/Sz / b / 34. (M) Anguinae indet., osteoderm, external view; GIUS 10–3796/Sz / b / 35. (N) Anura, vertebra fragment: N1, ventral, N2, dorsal view; GIUS 10–3796/Sz / b / 36. Scale bars: one mm. Note: attributions are tentative and based on limited diagnostic features.

### Extant excrements

For comparative analysis, more than 100 modern excrement samples were examined, supplemented by archival data from Brachaniec et al. (2022), Brachaniec et al. (2025). Taxa included:

-fish: leopard shark (*Stegostoma fasciatum*), brownbanded bamboo shark (*Chiloscyllium punctatum)*, great barracuda (*Sphyraena barracuda*), jointed razorfish (*Aeoliscus strigatus*), Indonesian tiger perch (*Datnioides microlepis*);

-reptiles: spectacled caiman (*Caiman crocodilus*), common grass snake (*Natrix natrix*), king python (*Python regius*), tiger python (*Python molurus*), reticulated python (*Malayopython reticulatus*), common boa (*Boa constrictor*), king cobra (*Ophiophagus hannah*), Jamaican boa (*Chilabothrus subflavus*), Korean ratsnake (*Elaphe anomala*), Madagascar boa (*Acrantophis madagascariensis*), European viper (*Vipera berus*), Komodo dragon (*Varanus komodoensis*), noble iguana (*Iguana delicatissima*), brown turtle (*Manouria emys*), Mediterranean tortoise (*Testudo hermanni*), steppe tortoise (*Testudo horsfieldii*), Indian star tortoise (*Geochelone elegans*), Spanish pond turtle (*Mauremys leprosa*), Nile soft shell turtle (*Trionyx triunguis*);

-birds: house sparrow (*Passer domesticus*), city pigeon (*Columba livia*), white-tailed eagle (*Haliaeetus albicilla*), crested partridge (*Rollulus rouloul*);

-mammals: Seba’s short-tailed bat (*Carollia perspicillata*), brown hare (*Lepus europeaus*), European mole (*Talpa europaea*), guinea pig (*Cavia porcellus*), Swinhoe’s striped squirrel (*Tamiops swinhoei*), European beaver (*Castor fiber*), African lion (*Panthera leo*), cheetah (*Acinonyx jubatus*).

This comparative dataset provided a framework for evaluating morphological and compositional features of the coprolites.

### Optical and SEM microscopy studies

Analysis of the specimen GIUS 10–3796/Sz/c/11 (Bełchatów) revealed a two-layered matrix: the outer zone composed of fine iron oxides and hydroxides, and the central zone consisting of massive, granular siderite (Figs. 5A, 5C, 5E, 5G, 5H). Other specimens lacked this distinct layering, instead showing siderite grains coated with a thin veneer of iron oxides and hydroxides (Figs. 5B, 5D, 5F, 5I). Hair-like inclusions possibly organic/biogenic in origin were observed in coprolite GIUS 10–3796/Sz/c/1 (Figs. 2M, 2N).

**Figure 5:**
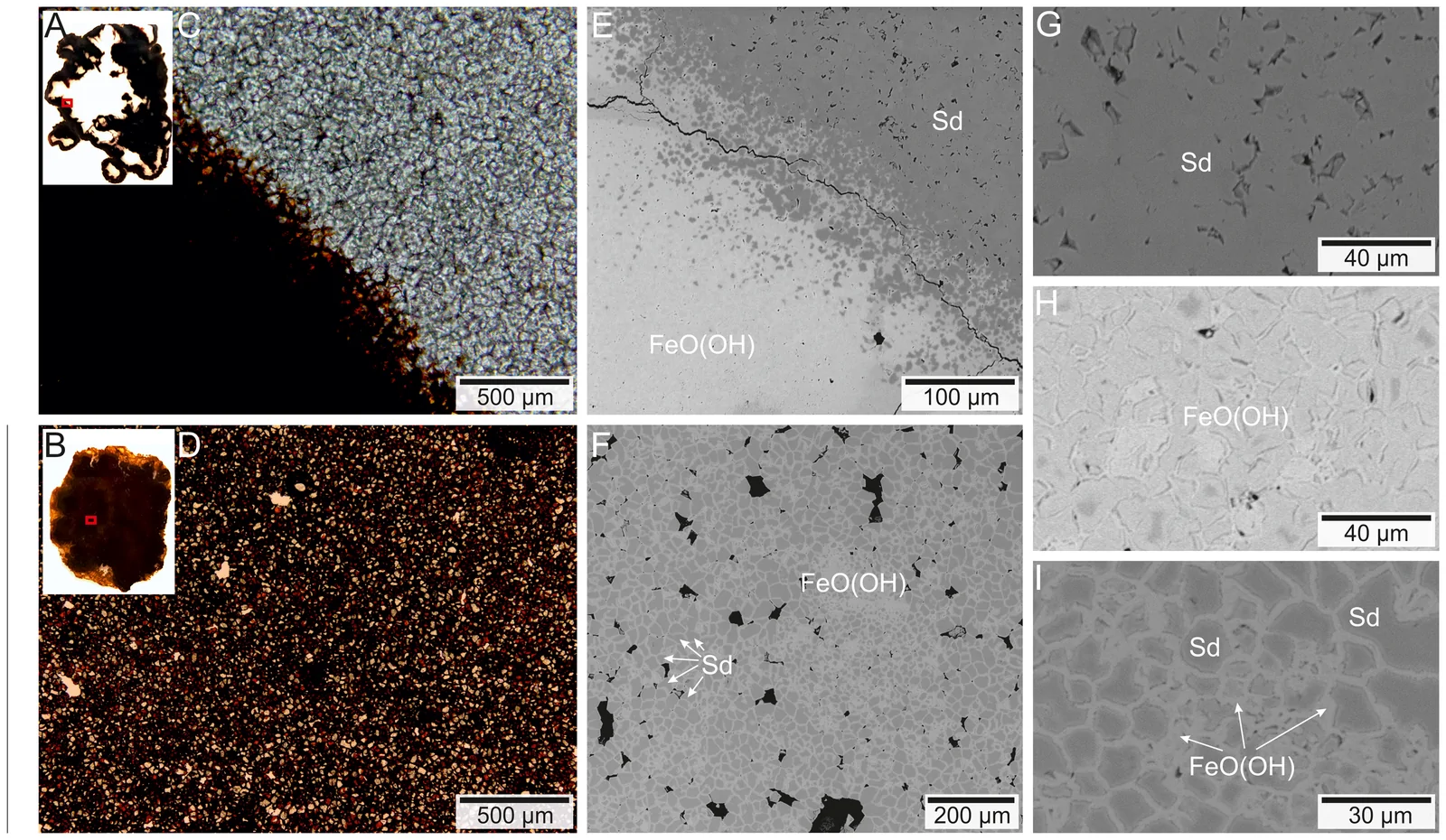
Thin-section and SEM images of probable coprolite specimens from Bełchatów. (A, B) Thin sections of coprolites; (C, D) Coprolite matrix under optical microscopy; (E, F) BSE images of coprolite matrix; (G, H) SEM close-up of siderite and iron oxides/hydroxides in specimen GIUS 10–3796/Sz/11; (I) SEM close-up of mixed siderite and iron oxides/hydroxides in coprolite matrix. Mineral abbreviations: Sd –siderite, FeO(OH) –iron oxides and hydroxides.

## Discussion

The current mineralogical composition of the ferruginous, excrement-shaped masses from central Poland does not unequivocally confirm their interpretation as coprolites. However, substantial post-depositional alteration, most likely advanced diagenetic overprinting, provides a plausible explanation (Broughton, Simpson & Whitaker, 1977; Broughton, Simpson & Whitaker, 1978; Broughton, 2017). Comparable ferruginous morphologies from Miocene deposits in southwestern Washington (USA) were interpreted by Seilacher et al. (2001) as products of secondary chemical modification involving oxidizing groundwater. Such redox-controlled “roll-front” processes (Goldhaber et al., 1987; Harris & King, 1993) can dissolve original biogenic components (*e.g.*, phosphates and calcite, typically associated with faecal matter or bone) while simultaneously facilitating the precipitation of iron-rich carbonate such as siderite or ferroan dolomite.

The geochemical and microstructural features of the analyzed specimens, including abundant internal voids and the absence of clay minerals, are consistent with this diagenetic scenario. Their lithostratigraphic context (at the interface between a mud-clay horizon and an overlying permeable sand unit), likely facilitated subsurface fluid movement, enhancing mineral replacement. Such processes are well documented in lignite-bearing basins, where phosphate phases are commonly substituted by iron-bearing minerals.

Importantly, the absence of phosphate does not preclude a biogenic origin. Triassic coprolites from Brazil show near-complete replacement of apatite by calcite (Francischini, Dentzien-Dias & Schultz, 2018). Low phosphate levels may also reflect herbivorous diets, as herbivores excrete less phosphate-rich material than carnivores (Chin & Kirkland, 1998; Fiorelli et al., 2013; Bajdek et al., 2016). Therefore, geochemistry alone cannot be served as a definitive criterion for excluding a faecal origin. It appears that morphological and size comparisons with modern feces constitute the best method for identifying potential coprolites. Morphological evidence provides stronger support for a coprolitic interpretation. The specimens exhibit consistent elongation, tapering ends (Figs. 2A–2F), and surface striations (Fig. 2B). These striations, both longitudinal and transverse, differ from diagenetic cracks and may reflect biological processes, though interpretations such as sphincter contraction impressions remain tentative. Microscopic analysis revealed filamentous structures (Figs. 2M, 2N) that may represent organic inclusions, though mineral or diagenetic origins cannot be entirely excluded. Similar filamentous structures have been interpreted as potential hair (*e.g.*, Pesquero et al., 2011; Dentzien-Dias et al., 2018; Brachaniec et al., 2022). Some of similar structures could also be interpreted as possible traces of biological activity, such as filamentous cyanobacteria (Dentzien-Dias et al., 2026) or possible parasites (Brachaniec et al., 2015).

Excrement morphology exhibits considerable intra- and interspecific variability, influenced by diet, physiology, and environmental conditions. The presence of filamenteous-like structures resembling hairs within the Bełchatów specimens may suggest the consumption of small vertebrates.

The described remains differ significantly from the known teeth of herbivorous fish from Bełchatów, specifically the pharyngeal teeth of cyprinids, whose diet also partly consisted of malacofauna. Reptilian remains are indisputably present among the identified specimens. Certain identifications pertain primarily to crocodile teeth of the genus *Diplocynodon* sp. (Figs. 3B1–3B2; 4O); nearly identical teeth have been identified from Subpiatră, Romania (Venczel, 2007). The teeth are labiolingually flattened, slightly curved, and feature a well-developed mesiodistal edge. The remains shown in Figs. 3N1–3N3 represent another group of reptiles that can be confidently identified. Pleurodont dentition-characterized by teeth attached to the inner surface of the upper and lower jaws-is found in snakes, lizards, and amphisbaenians. This allows us to rule out reptiles with different types of dentition, such as crocodiles, turtles, or tuataras. The only group with a similar form of dentition is the amphibians; however, the high margin of the jawbone and the absence of traces of pedicellate teeth tend to rule this out. Due to the fragmentary nature of this specimen, we decided to classify it as a probable squamate. Another type of diagnostic remains are osteoderms from anguid lizards. A distinctive feature is a narrow medial ridge, from which numerous tubercles and depressions radiate outwards. Similar plates have been described from the Miocene of Germany (Vasilyan et al., 2022; fig. 16). The bony plates of frogs (Fig. 4G1) are similar in form; however, their distribution on the body is largely restricted to the skull, and they lack such a distinct central ridge. Another specimen was identified as reptilian tooth (Fig. 3A) and initially classified as belonging to a small varanoid, due to the presence of radiating structures resembling plicadentine and a wide, conical pulp cavity. We interpret this tooth as belonging to a reptile with thecodont dentition, possibly a juvenile crocodile. Teeth presented in Figs. 4F1–4F2, 4H1–4H2, 4I1–4I2, and 4J1–4J3 have no direct analogy in the literature; the teeth are blunt (Figs. 4F, 4H, 4J), massive and likely adapted for crushing snails, which points toward snakes or lizards, with more or less distinct lateral edge (Fig. 4I). The presence of enamel, wide canals at the tooth base, the absence of roots, and lateral notches at the base may suggest a pleurodont or thecodont type of dentition.

Comparisons with modern faeces highlight morphological similarities to squamates. The closest resemblances were observed with the noble iguana (*Iguana delicatissima*; Figs. 2G, 2J) and the Madagascar boa (*Acrantophis madagascariensis*; Figs. 2H, 2I), both showing elongation and compression. Additional similarity was noted in the Jamaican boa (*Chilabothrus subflavus*; Fig. 2K). In contrast, turtle faeces, although sometimes elongated, lack comparable compression and differ morphologically (Fig. 2L). These comparisons suggest a squamate origin, though diagenetic alteration and morphological variability warrant caution.

The taphonomic pathways leading to sideritic masses interpreted as coprolites remain complex. Proposed models include early diagenetic mineralization, roll-front processes, and iron mineral replacement (Seilacher et al., 2001; Broughton, 2017; Broughton, 2021; Mustoe, 2025; Rey et al., 2026). These models emphasize different mechanisms of mineral precipitation and organic matter transformation, yet each has limitations, and none fully explains all observed features.

It has been demonstrated that siderite concretions may form around organic remains without replacement (Krzykawski et al., 2014; Tripp et al., 2025; Raiswell & Fisher, 2000), while partial or complete replacement is also documented (Briggs, 2003; Mustoe, 2025; Rey et al., 2026). Such variability reflects microenvironmental differences in redox gradients, microbial activity, and sediment permeability (Berner, 1981; Raiswell & Fisher, 2000).

The specimens studied here share features with previously described Miocene sideritic coprolites from Poland, traditionally interpreted as bromalites (Brachaniec et al., 2022; Brachaniec et al., 2025). Their internal structure, inclusions, association with vertebrate, remains support a coprolitic origin, though abiotic scenarios cannot be entirely excluded. In addition next comparisons with modern feces, experimental taphonomy also appears to be a promising method for identifying potential coprolites from older sediments. It would allow for the observation of how water affects shape the preservation of fecal, as well as the morphological changes occurring within the sediment as the specimen undergoes varying degrees of compaction. Overall, the evidence supports a coprolitic origin for the Bełchatów specimens, though the taphonomic pathways are complex and multifactorial. A cautious interpretative framework is therefore adopted, acknowledging both the strengths and limitations of the available data.

### Miocene palaeoenvironment and producers of Bełchatów coprolites

Fossil plant assemblages from Bełchatów are associated with slow-flowing rivers, wetlands, and oxbow lakes (Worobiec, 1995; Worobiec, 2003; Worobiec & Szynkiewicz, 2007; Worobiec & Worobiec, 2008). Associated ichthyofauna shows low diversity, suggesting environmental stress (Kovalchuk et al., 2019; Fig. 6). The palaeoflora is dominated by palaeotropical and thermophilic taxa, including conifers (*Glyptostrobus*, *Tetraclinis*) and angiosperms (*Daphnogene*, *Laurophyllum*, *Smilax*), with pollen from Arecaceae, Mastixiaceae, Meliaceae, and Sapotaceae. Climatic conditions were subtropical and humid (Köppen–Geiger Cfa type; Kottek et al., 2006), with mean annual temperatures of 16.8–17.8 °C. Additional indicators include terrestrial fungi and mollusks typical of warm, humid environments (Worobiec & Worobiec, 2017; Salamon, Bugajski & Degórski, 2025). Vertebrate assemblages support a wetland ecosystem with permanent water (Kowalski, 1993a; Kowalski, 1993b; Kowalski, 1997; Rzebik-Kowalska, 1993; Rzebik-Kowalska, 1994; Rzebik-Kowalska, 1996; Rzebik-Kowalska, 2005; Nadachowski, 2001). Reptilian remains, including squamates and crocodylians (*e.g.*, *Diplocynodon*; Górka et al., 2025), indicate suitable ecological niches for coprolite producers. However, direct taxonomic assignment of the studied specimens remains uncertain, due to the lack of comparable material in the available literature-specifically plates and figures illustrating holotype specimens. Most studies from this period concerning terrestrial, freshwater, and brackish environments are limited to only a few localities, particularly regarding Poland. While certain animal remains-such as osteoderms, pharyngeal teeth of cyprinid fishes and rodent teeth are relatively well-represented, others have only marginal attention due to low fossilization potential or rarity.

**Figure 6:**
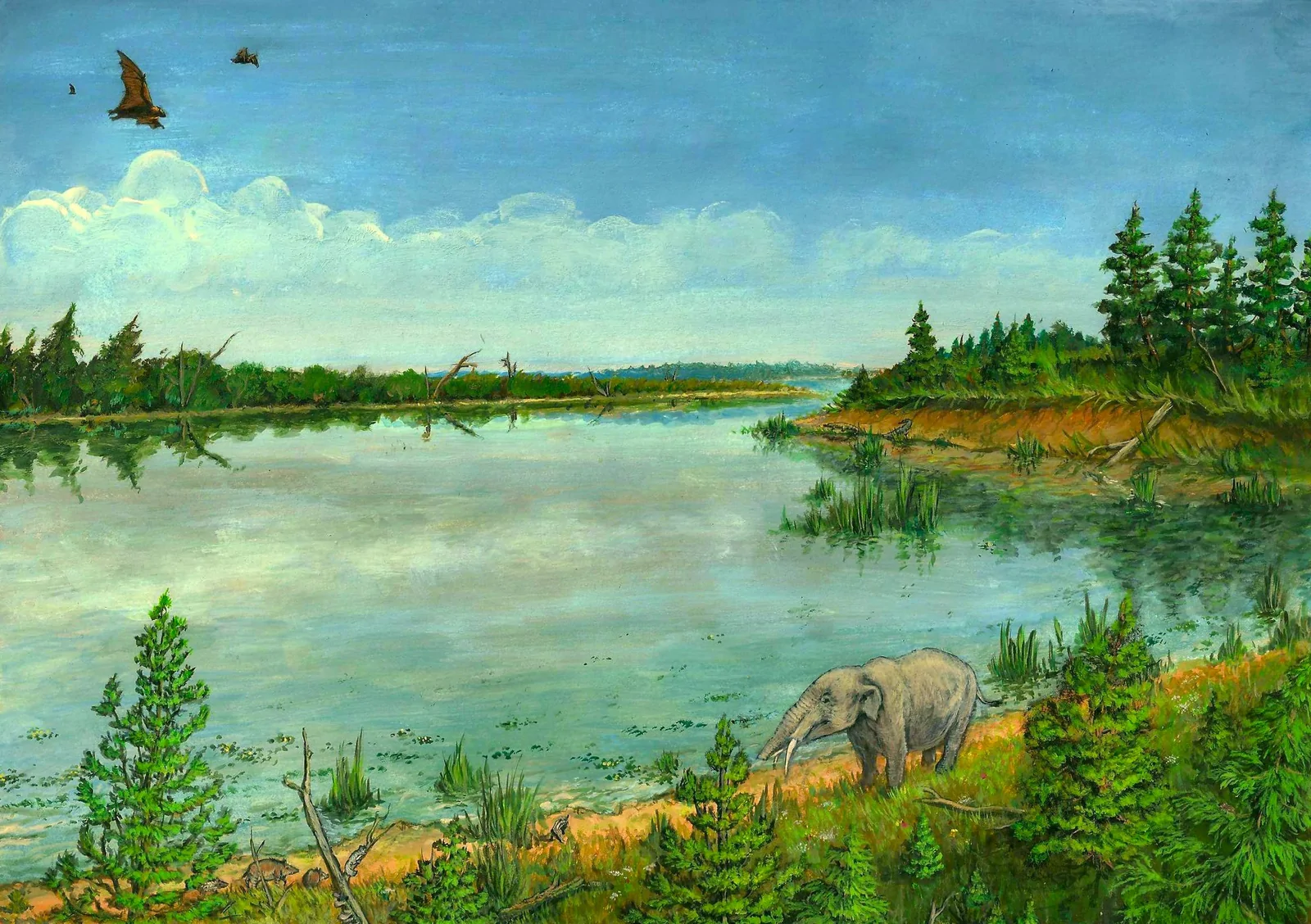
Artistic vision of the Miocene palaeoenvironment in Bełchatów (drawn by BC).

## Conclusions

The ferruginous, excrement-shaped masses recovered from the Miocene deposits of the Kleszczów Graben in central Poland are best interpreted as probable coprolites, most likely produced by squamates (lizards and/or snakes). Distinctive morphological features, including elongation, compression, tapering ends, and fine surface striations, support this interpretation. The occurrence of filamentous, hair-like structures provides additional, though cautious, evidence for a biogenic origin, potentially reflecting the consumption of small vertebrates.

Comparative analyses with modern reptilian faeces further support a squamate origin, while turtles and abiotic processes appear less consistent with the observed features. Although mineralogical composition reflects strong diagenetic alteration, it does not preclude a biological origin and highlights complex preservation pathways involving iron-rich groundwater.

The faunal assemblage, together with sedimentological and paleobotanical data, indicates a subtropical, humid Miocene ecosystem with diverse vertebrate communities. These findings provide insights into trophic interactions and underscore the value of coprolites for reconstructing ancient food webs. Overall, the combined morphological, microstructural, and faunal evidence supports a coprolitic origin, while acknowledging remaining uncertainties.

##  Supplemental Information

10.7717/peerj.21755/supp-1 Supplemental Information 1 Characteristics of the coprolites examined from the Bełchatów area, catalogued under registration numbers GIUS 10–3796/Sz/c/1-22

10.7717/peerj.21755/supp-2 Supplemental Information 2 List of vertebrates collected in Miocene sediments of the Bełchatów area

## Additional Information and Declarations

### Data Availability

The following information was supplied regarding data availability: The data is available in the Supplemental Files. The data is available at Zenodo: Brachaniec, T. (2026). High-resolution photos of vertebrate remains from Bełchatów [Figure]. Zenodo. https://doi.org/10.5281/zenodo.19552055
All specimens are curated at the Institute of Earth Sciences, Faculty of Natural Sciences, University of Silesia in Katowice, Poland, where they are stored under the registration summary number GIUS 10–3796/Sz/c/1-22.
