# Supplementary material for: Reassessment of Miocene putative coprolites from Poland suggests a biogenic origin and probable squamate (reptilian) producers
Source: PeerJ. 2026 Sep 29;14:e21755. doi: 10.7717/peerj.21755 (PMC13632681; doi:10.7717/peerj.21755)
Supplement: Supplementary file 1 [file peerj-14-21755-s001.docx]

**Supplementary Table 1**. Characteristics of the coprolites examined from the Bełchatów area, catalogued under registration numbers GIUS 10–3796/Sz/c/1-22 (see Materials and Methods).

| **Specimen** | **Dimensions – length × width (mm)** | **Characteristics** |
| --- | --- | --- |
| 1 | 142×44 | Elongated; light brown; GIUS 10–3796/Sz/c/1; *Figure 2A* |
| 2 | 61×13 | Less squeezed; pale orange; GIUS 10–3796/Sz/c/2; *Figure 2B* |
| 3 | 96×38 | Less squeezed; pale orange; GIUS 10–3796/Sz/c/3; *Figure 2C* |
| 4 | 35×12 | More squeezed; burgundy; GIUS 10–3796/Sz/c/4; *Figure 2 D* |
| 5 | 51×14 | Elongated; pale orange; GIUS 10–3796/Sz/c/5; *Figure 2E* |
| 6 | 84×43 | More squeezed; burgundy; GIUS 10–3796/Sz/c/6; *Figure 2F* |
| 7 | 53×16 | Elongated; pale orange; GIUS 10–3796/Sz/c/7 |
| 8 | 40×18 | More squeezed; light brown; GIUS 10–3796/Sz/c/8 |
| 9 | 72×29 | Less squeezed; pale orange; GIUS 10–3796/Sz/c/9 |
| 10 | 59×20 | Elongated; light brown; GIUS 10–3796/Sz/c/10 |
| 11 | 66×25 | Less squeezed; light brown; GIUS 10–3796/Sz/c/11 |
| 12 | 54×30 | More squeezed; greenish red; GIUS 10–3796/Sz/c/12 |
| 13 | 42×17 | Less squeezed; greenish red; GIUS 10–3796/Sz/c/13 |
| 14 | 75×22 | Elongated; burgundy; GIUS 10–3796/Sz/c/14 |
| 15 | 48×19 | Less squeezed; pale orange; GIUS 10–3796/Sz/c/15 |
| 16 | 66×31 | More squeezed; pale orange; GIUS 10–3796/Sz/c/16 |
| 17 | 61×23 | Less squeezed; burgundy; GIUS 10–3796/Sz/c/17 |
| 18 | 58×21 | More squeezed; light brown; GIUS 10–3796/Sz/c/18 |
| 19 | 80×26 | Elongated; greenish red; GIUS 10–3796/Sz/c/19 |
| 20 | 63×24 | Less squeezed; pale orange; GIUS 10–3796/Sz/c/20 |
| 21 | 51×18 | More squeezed; burgundy; GIUS 10–3796/Sz/c/21 |
| 22 | 88×29 | Elongated; pale orange; GIUS 10–3796/Sz/c/22 |
| 23 | 74×20 | Elongated; pale orange; GIUS 10–3796/Sz/c/23 |
