# Supplementary material for: Reassessment of Miocene putative coprolites from Poland suggests a biogenic origin and probable squamate (reptilian) producers
Source: PeerJ. 2026 Sep 29;14:e21755. doi: 10.7717/peerj.21755 (PMC13632681; doi:10.7717/peerj.21755)
Supplement: Supplementary file 2 [file peerj-14-21755-s002.docx]

**Supplementary Table 2**. List of vertebrates collected in Miocene sediments of the Bełchatów area.

| **Order/family/genus** | **Taxon** |
| --- | --- |
| Squamata/Anguidae/Anguinae | Anguinae indet.; *Figure 4C, 4M* |
| Anura | Anura indet.; *Figure 3E1-2, 4L1-2, 4N* |
| Anura/Pelobatidae/*Pelobates* | *Pelobates* sp.; *Figure 4G1-2* |
| Chiroptera/Vespertilionidae*/Myotis* | *Myotis* sp.; *Figure 3C1-2* |
| Eulipotyphla/Erinaceidae | Erinaceidae indet.; *Figure 3D1-2, 3L1-3* |
| Erinaceomorpha/Dimylidae/?Plesiodimylinae | ?Plesiodimylinae indet.; *Figure 3P, Q* |
| Eulipotyphla/Soricidae/*Plesiosorex* | *Plesiosorex germanicus*; *Figure 3T1-T3* |
| Crocodylia indet. | Crocodylia indet.; *Figure 3B1-2* |
| Lagomorpha | Lagomorpha indet.; *Figure 3H1-2* |
| Eulipotyphla/Talpidae/*Desmanella* | *Desmanella* sp.; *Figure 3I1-2* |
| Eulipotyphla/Talpidae/*Desmanella* | *Desmanella* cf. *engesseri*; *Figure 3S1-3* |
| Eulipotyphla/Talpidae/*Desmanella* | *Desmanella* cf. *stehlini*; *Figure 3O1-3, 3U1-3* |
| ?Reptilia | Reptilia indet.; *Figure 4F1-2, 4H1-2, 4I1-2, 4J1-3* |
| Rodentia | Rodentia indet.; *Figure 3F1-3, 3J1-2, 3K1-3* |
| Rodentia/Eomyidae/*Keramidomys* | *Keramidomys* sp.; *Figure 3M1-2* |
| Esociformes/Esocidae/*Esox* | *Esox* sp.; *Figure 4E1-2* |
| Eulipotyphla/Talpinae | Talpinae indet.; *Figure 3R1-2, 3V1-4* |
| ?Squamata indet. | ?Squamata indet.; *Figure 3N1-3* |
| Reptilia indet. | Reptilia indet.; *Figure 3A1-2* |
| Crocodylia/ Diplocynodontidae/Diplocynodon | *Diplocynodon sp.*; *Figure 4O* |
| ?? | *??; Figure 4D1-2* |
